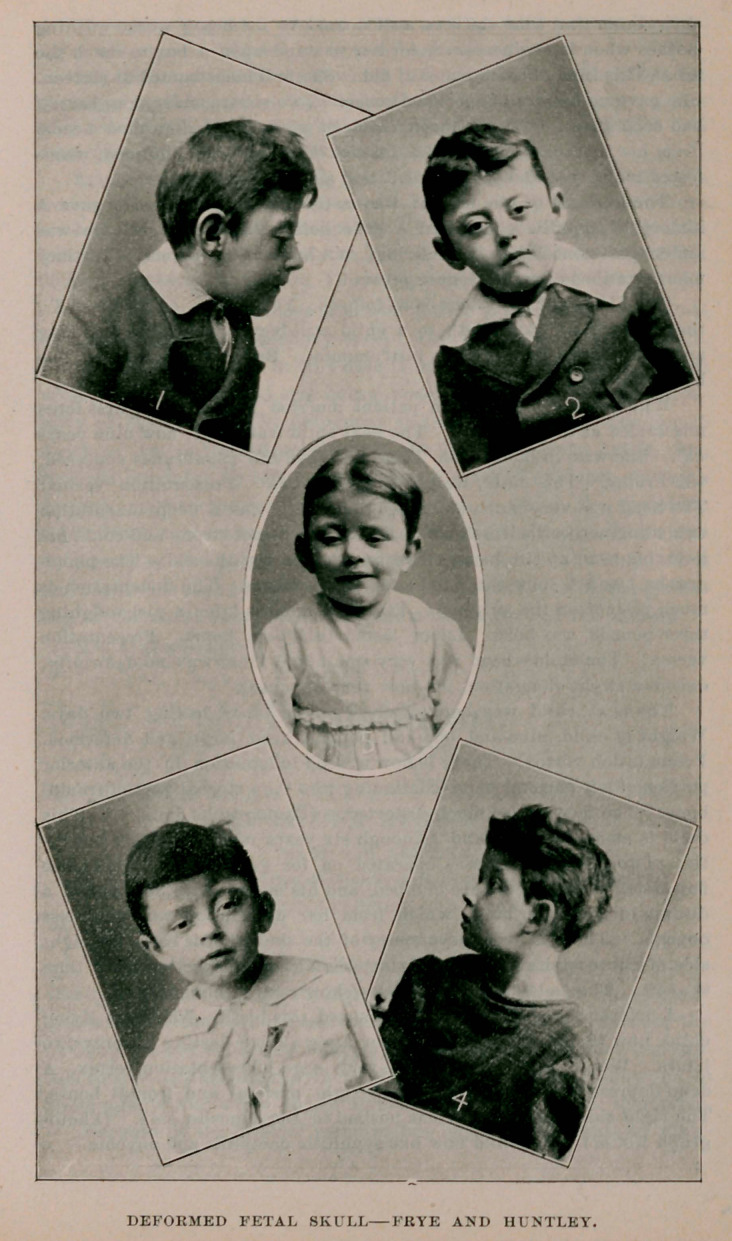# Three Cases of Deformed Fetal Skull

**Published:** 1896-06

**Authors:** Maud J. Frye, Mary M. Huntley

**Affiliations:** 224 Allen Street; 224 Allen Street


### Clinical Report.

### THREE CASES OF DEFORMED FETAL SKULL.

Reported by MAUD J. FRYE, M. D., and MARY M. HUNTLEY, M. D.

In December, 1895, the writers attended in confinement a woman, the mother of five children, three of whom had been born with deformed heads. She had been attended each time by the same midwife, to whom we are indebted for an account of the labors and the presentations as we give them. No history of positions was obtained.

The patient, Mrs. X., was a French Canadian, 35 years old. She gave a history of good health. When nine years old she began working

DEFORMED FETAL SKULL—FRYE AND HUNTLEY.

out. From that time she was accustomed to do heavy work, washing clothes when it was necessary for her to stand upon a box to reach the tub. This kind of work she still did. She first menstruated at sixteen. She gave no history of specific disease. Two sisters older than herself had each given birth to three children with badly deformed heads. From her mother we obtained a history of rickets in childhood, manifested in delayed and difficult dentition and late walking.

The husband of the patient was a German, aged 34. He gave a history of syphilis contracted a year before his marriage. He was under treatment before his marriage, but had not been since. Tertiary manifestations of syphilis were present.

The history of the labors is as follows: At the age of 23 Mrs. X., then unmarried, gave birth to a child still-born. The labor was long and difficult and delivery was instrumental. Breech presentation. The head was not deformed.

When 25 years of age the patient married Mr. X. The first fetus miscarried at three months. The next child was a boy, now nine years old. She was in labor from Tuesday, when the membranes ruptured, till Friday. The child weighed ten pounds. Presentation vertex. The head was very much elongated and there was a deep indentation extending across the forehead. The baby was not strong and could not hold his head up till he was seven or eight months old. The photographs 1 and 2 show the head as it now appears. The indentation is much plainer on the original. Eighteen months later a girl weighing three pounds was born. Labor lasted thirty-six hours. Presentation vertex. The child's head was very small and there was no deformity, except a slight elongation, not now very apparent.

The next child was born after a severe labor, lasting two days. Weight of child, nine and one-half pounds, head large and deformed. Presentation vertex. There is now a deep indentation in the anterior portion of left parietal bone, continuing into the external part of frontal bone. The face is also much distorted. (Photographs 3 and 4.) This child is mentally weak, and although six years of age can talk but little. Two years ago he was operated on for genu varum. He is also flat-footed. His fingers are clubbed, and his mother says he has had a disease of the finger nails, which from her description was doubtless onychia. There is great asymmetry of the skull in this case, the right side of the occipital and the right parietal bone projecting more than the left. The ends of the long bones show rachitic changes.

A miscarriage at five months followed this birth. The next living child, now three years old, was born after a labor lasting seventy-two hours. Weight six pounds. Head very large. Presentation vertex. A deep depression is now seen on the right parietal and frontal bones. The right side of the face seems pushed to the opposite side. (Photograph No. 5.) The child now has syphilitic dactylitis and onychia.

The succeeding labor was not difficult. The child was a boy weighing seven pounds, the head being small and not deformed.

The sixth and last labor, at which time we saw the mother, was not prolonged and was of no special interest. Presentation vertex. The head was small, with a slight grooving of the forehead, which has since partially disappeared. Diameters of head normal.

Examination of the patient before the birth of the child showed a woman of medium height and weight. Slight rotary-lateral curvature of the spine with tilting of the pelvis. Pelvic measurements: Between ant. sup. spinous processes of the ilia, 30 cm.; between crests of ilia, 28.5 cm.; external conjugate, 17 cm.; internal conjugate, 9 cm.

The history of the case and the pelvic measurements exclude all forms of pelvic deformity except the flattened rachitic pelvis. The pelvic contraction in this case is not great, the birth of a child with a head of normal size being possible with little or no deformity of the skull. The deformity in these cases seems due not so much to the contracted pelvis as to the unusually large heads, due, may we not infer, to congenital rickets? marked evidence of which disease exists in the case of one child, and to which the syphilitic taint from the father would predispose the children. From the deformity of the pelvis present with the account of the long labors one concludes that the delay occurred at the pelvic brim, the grooving of the skull being caused by the promonitory of the sacrum.

224 ALLEN STREET.

## Figures and Tables

**Figure f1:**